# Boosting Productivity for Advanced Biomanufacturing by Re-Using Viable Cells

**DOI:** 10.3389/fbioe.2023.1106292

**Published:** 2023-02-16

**Authors:** Lucas Nik Reger, Martin Saballus, Jens Matuszczyk, Markus Kampmann, Rene H. Wijffels, Dirk E. Martens, Julia Niemann

**Affiliations:** ^1^ Corporate Research, Sartorius, Göttingen, Germany; ^2^ Bioprocess Engineering, Wageningen University, Wageningen, Netherlands; ^3^ Faculty of Biosciences and Aquaculture, Nord University, Bodø, Norway

**Keywords:** CHO cell culture, process intensification, fluidized bed centrifuge, intermediate 9 harvest, monoclonal antibodies

## Abstract

Monoclonal antibodies (mAb) have gained enormous therapeutic application during the last decade as highly efficient and flexible tools for the treatment of various diseases. Despite this success, there remain opportunities to drive down the manufacturing costs of antibody-based therapies through cost efficiency measures. To reduce production costs, novel process intensification methods based on state-of-the-art fed-batch and perfusion have been implemented during the last few years. Building on process intensification, we demonstrate the feasibility and benefits of a novel, innovative hybrid process that combines the robustness of a fed-batch operation with the benefits of a complete media exchange enabled through a fluidized bed centrifuge (FBC). In an initial small-scale FBC-mimic screening, we investigated multiple process parameters, resulting in increased cell proliferation and an elongated viability profile. Consecutively, the most productive process scenario was transferred to the 5-L scale, further optimized and compared to a standard fed-batch process. Our data show that the novel hybrid process enables significantly higher peak cell densities (163%) and an impressive increase in mAb amount of approximately 254% while utilizing the same reactor size and process duration of the standard fed-batch operation. Furthermore, our data show comparable critical quality attributes (CQAs) between the processes and reveal scale-up possibilities and no need for extensive additional process monitoring. Therefore, this novel process intensification strategy yields strong potential for transfer into future industrial manufacturing processes.

## 1 Introduction

Recombinantly expressed biotherapeutics, such as monoclonal antibodies (mAb), hormones, cytokines, and vaccines, have gained attention in the pharmaceutical industry during recent years (Walsh, 2018). Especially, novel therapies based on mAbs have become a key interest for clinical application and biomanufacturing since the first clinical approval in 1986 (Orthoclone OKT3^®^). Since then, countless clinical trials have highlighted the distinct advantages of these biotherapeutics, such as flexible adjustment to application, low toxicity, high specificity, and suitable *in vivo* half-life (Imai and Takaoka, 2006; Ecker et al., 2015; Castelli et al., 2019). As a result, more than a 100 mAb-based therapeutics have been approved by the US Food and Drug Administration (FDA) by today, and many more are in clinical development, proving the still increasing interest in these biopharmaceuticals (Gillespie, 2021).

Therapeutic antibodies are characterized by complex post-translational modifications to ensure bioactivity and low toxicity of the proteins. Therefore, mammalian cell lines like Chinese hamster ovary (CHO) cells are utilized for the expression of these complex biomolecules instead of microbial hosts (Jayapal et al., 2007; Kim et al., 2012). In contrast to small-molecule therapies, production costs are rather high and antibody-based immunotherapies are still very costly (Hernandez et al., 2018; Donini and Marusic, 2019). To lower costs, novel innovative intensification strategies are necessary.

Discontinuous process formats like fed-batch (FB) are state of the art for large-scale production of mAbs due to their robustness, simplicity, and reproducibility (Birch and Racher, 2006; Huang et al., 2010). The process can be divided into three consecutives culture stages: the logarithmic phase with exponential cell growth, the stationary phase without cell growth, and the death phase with decreasing cell viability usually due to an increasing number of apoptotic cells. During an FB process, where nutrient limitation is prevented, the transition between these different stages is commonly triggered by the accumulation of inhibitory molecules such as growth inhibitors, by-products, and fragments of lysed cells, which ultimately limit the overall productivity and time span of the process (Ahn and Antoniewicz, 2011; Carinhas et al., 2013; Mulukutla et al., 2019). Since 1990, product titers in discontinuous formats have improved from 0.1 g/L up to 5 g/L, mainly by optimization of cell lines, media systems, process control, and switch from batch to the FB mode (Jayapal et al., 2007; Huang et al., 2010). Furthermore, different strategies have been developed to enhance the productivity of the cells, such as the addition of specific molecules that enhance the productivity, increased osmolarities, as well as shifts in temperature or pH during the stationary phase which allow to sustain cell viability and productivity for an extended period of time (Kumar et al., 2007; Hogiri et al., 2018; Alhuthali et al., 2021; Schulze et al., 2021). However, the key limitation of discontinuous operations is the accumulation of cell-derived inhibiting molecules as well as nutrient limitation in the media, which consecutively limits the volumetric productivity of the process (Pereira et al., 2018).

To bypass disadvantageous characteristics of discontinuous process formats, alternative process strategies have been pursued based on constant media exchange. These perfusion cell culture systems enable further proliferation of the cells by exchange of the media matrix and consequently the removal of inhibitory molecules along with the supply of fresh nutrients (Chen et al., 2018). In order to constantly remove the cell-free media fraction, cell retention devices have been developed, with the most common systems being based on alternating tangential flow (ATF) or tangential flow filtration (TFF) (Wang et al., 2017; Bielser et al., 2018). By employing a bleed system to regulate the viable cell concentration, a pseudo-steady-state can be reached to prevent nutrient limitation and the concentration of cellularly derived molecules, thus allowing for continuous cultivation (Dowd et al., 2003). Thus, perfusion cell culture enables increased volumetric productivity and plant utilization in comparison to discontinuous processes (Ozturk, 2014; Konstantinov and Cooney, 2015). However, these continuous cultivations require large amounts of media—up to several reactor volumes per day—large membrane devices for cell retention, and complex process monitoring. This increased media consumption is one of the main cost drivers in the perfusion process (Wang et al., 2017; Bielser et al., 2018).

All of these hybrid processes utilize a filter system to continuously exchange the spent media in the reactor. Thereby, filter systems show distinct drawbacks like a slow exchange rate which leads to the back mixing of new supplied media as well as clogging and fouling (Shirgaonkar et al., 2004). One emerging technology in this field is the fluidized bed centrifuge (FBC), which allows for fast and aseptic separation of cells from the supernatant as well as their washing and concentration under mild process conditions. The functional principle of the FBC relies on cell capture resulting from counteracting centrifugal forces and flow forces while low-molecular-weight particles like spent media and mAb can flow through. This system was first developed as cell clarification equipment, showing high recovery rates for mAb and cells (Saballus et al., 2021). However, the non-invasive FBC separation of cells and medium has also been postulated to be applicable during the upstream processing to allow for a complete media exchange mid-process. The general concept of returning the washed cells into the cell culture system has been proposed over 10 years ago (Mehta et al., 2010), while a concept including two bioreactors operated in parallel, where the cell broth is processed *via* the FBC system and transferred into the second bioreactor, has been described in the study by Jacob Arthur Tijsterman (2015). However, proof-of-concept data for these process concepts are still missing.

**FIGURE 1:**
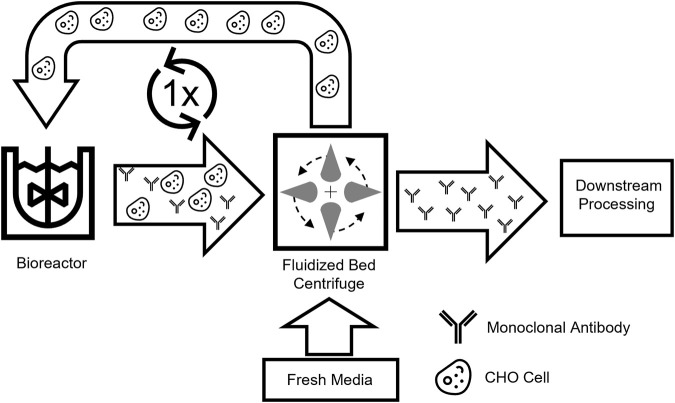
Schematic overview of the intermediate harvest process. Cell broth with secreted products is harvested from a bioreactor and separated by a single-use fluidized bed centrifuge. Afterward, the washed cells can be reintroduced into the production bioreactor and utilized in a new process.

Within this work, we present a novel process scenario in which only one bioreactor is employed and a media exchange is applied to intensify a common fed-batch operation. This new intensified process concept, based on the beneficial media exchange provided by the FBC, was developed to enable higher volumetric productivity while bypassing excessive media usage and the need for extensive process control. This novel process is based on an intermediate harvest (IH) step where, subsequent to a first cultivation phase, the product is harvested while cells are being washed with fresh media and returned to the same production reactor for a second production phase (Figure 1). The new intensified process was tested in a small-scale screening system to investigate the impacts on the cultivation and consecutively scaled-up and further optimized. Overall, an intensified process with beneficial characteristics in productivity and media consumption should be developed with scale-down options.

## 2 Materials and methods

### 2.1 Cell culture

#### 2.1.1 Cell line, media, and seed train

A commercially available Chinese hamster ovary (CHO-DG44) cell line (Sartorius Stedim Cellca) stably expressing mAbs was used as an industry-relevant model system. Prior to n-stage cultivation, the cells were thawed and passaged in a similar scheme to secure consistency between experiments. All cultivations were conducted with the same chemically defined 4Cell^®^ SmartCHO media system (Sartorius Stedim Biotech) including a basal medium (PM) and two feeding solutions (FMA and FMB).

#### 2.1.2 Small-scale screening

The concept was developed at small scale by use of the multi-parallel-bioreactor system Ambr15 (Sartorius Stedim Biotech). Three different time points of IH were investigated: the exponential phase at day 6, stationary phase at day 9, and death phase at day 11, which are representative for the different growth phases of the used CHO cells in a standard FB process. Each vessel was inoculated with 0.3 × 10^6^ cells/mL and a working volume of 13 mL. The temperature was controlled at 36.8°C and the pH set point was set to 7.1 by CO_2_ sparging. The homogenization of the bioreactors was ensured by a stirrer set point of 1,300 rpm, and the DO was controlled at 40% by O_2_ sparging. A fixed-bolus feeding by volume percentage with decreasing values overtime was conducted to mimic large-scale cultivation. To mimic the complete media exchange at the specified IH time points (days 6, 9, and 11), a modified version of the semi-perfusion system, implemented by Janoschek et al. (2019), was used. In brief, the vessel was removed from the Ambr15 system, the cell broth was transferred to a 15 mL Falcon (Sarstedt), and it was centrifuged (Centrifuge 3-30KS, Sigma) at 190 ×*g*, 3 min at room temperature (RT). The supernatant was sampled and stored at −20°C. The remaining cells were resuspended in the basal medium and centrifuged a second time, as described earlier. The cell pellet was then resuspended and reinoculated in the vessel with a mix of basal and feedmedia. The set point for termination was a viability of less than 70% in one of the triplicates.

#### 2.1.3 Scale-up

To test the feasibility and further optimize the IH process by incorporating the FBC for media exchange, the process was scaled-up to a 5 L UniVessel (UV) (Sartorius Stedim Biotech). The UV was inoculated with 0.3 × 10^6^ cells/mL, the temperature set point was 36.8°C, the pH was adjusted to 7.1 *via* CO_2_ sparing, and the DO was adjusted to 40% by supplying O_2_ to the basal gassing. The reactor was cultivated up to day 6, and the cell broth was processed by the FBC as described in 2.1.4. Subsequently, the washed cell broth was split and transferred to a suitable downscale model (Ambr250, Sartorius Stedim Biotech) to test different parameter sets within one trial. First, the feasibility experiment was investigated with reinoculation at similar cell densities as in the 5-L reactor at the moment of harvest to simulate the small scale, without the loss of cells (IH 25). Second, an optimization experiment was conducted with reinoculated cells at 40 × 10^6^ cells/mL after the IH (IH opt.); the background of this will be discussed in the results (see Section3.2). To handle the increased amounts of cells, IH processes were conducted with an adjustable feeding scheme based on a multiplication factor linked to cell density and the feeding was splitted to twice a day, and a daily glucose feed was added.

As in the last experiment, the optimized IH was conducted entirely in a 5-L reactor. Thereby, the process was started with an increased inoculation volume of 4 L and similar adjustments to the process feedings as described earlier.

#### 2.1.4 Cell separation

A Ksep^®^ 400 FBC system (Sartorius Stedim Biotech) was applied for sterile separation and washing of cells in the IH steps as well as for clarification in the final harvest step using a sterile Ksep^®^ 400 Harvest Clarification Consumable Kit for both processes.

To maintain sterility during the IH, all pre-sterilized receptions were connected by a BioWelder^®^ system (Sartorius Stedim Biotech) with the appropriated tubing to the FBC single-use consumables.

For both applications, IH and clarification, the FBC recipe for optimized cell washing with a cell broth loading flow rate of 100 mL/chamber was utilized (Saballus et al., 2021) with the exception of the washing buffer used; fresh cultivation medium (30°C–37°C) was used for IH to replace the spent medium. In the final harvest, pH 7.4 phosphate-buffered saline (PBS, chemicals supplied by Carl Roth) was used for washing out remaining mAb.

### 2.2 Analytics

The viable cell count (VCC), viability, and cell diameter were determined with the automatic cell counter, Cedex HiRes Cell Counter (Roche). A blood gas analyzer (ABL800 Basic, Radiometer) was used to measure pH as well as glucose and lactate concentration. Osmolality and ammonia levels were measured using a Nova Flex2 analyzer (Nova Biomedical). The remaining samples were centrifuged at 6,600 × *g*, 5 min at RT and stored at −20°C for further analysis.

The concentration of mAb was measured *via* size exclusion chromatography (SEC) utilizing a high-performance liquid chromatography (HPLC) system (Dionex UltiMate 3,000, ThermoFisher Scientific). The supernatant samples of each day were separated by a gel column (Yarra 3 μm SEC-3000, Phenomenex) at a flow rate of 1 mL/min. For quantification, a standard curve for each measurement set was conducted. The buffer solution contained 100 mM Na_2_SO_4_, 50 mM NaH_2_PO_4_, and 50 mM Na_2_HPO_4_. Product recoveries were calculated in accordance with the study by Saballus et al. (2021) taking into account the amount of biomass. The values for IVCC and cell-specific productivity (qP) were calculated according to the study by Janoschek et al. (2019).

Product quality was investigated by N-glycan determination. Therefore, supernatant samples were purified by ProtA columns (ProteinA HP SpinTrap, Cytiva) and desalted by Vivaspin ultrafiltration (Sartorius Stedim Biotech GmbH) with a 10 kDa molecular weight cutoff. Afterward, the glycans were detached and stained according to manufacturer protocol using the Glycan Release and Labeling Kit (PerkinElmer). The fluorescence labeled N-glycans were determined by LabChip GXII Touch24 (PerkinElmer).

To examine the amount of released host cell impurities in the process, broth host cell protein concentrations were determined using a commercial ELISA Kit (CYG-F550-1, Cygnus Technologies).

## 3 Results

In this study, we investigated the impact of a complete media exchange during a FB cultivation on cellular behavior and productivity using a commercially relevant CHO cell line producing mAbs. In contrast to a gradual exchange of media by utilization of classical cell retention devices in perfusion cell culture systems, a single complete and rapid media exchange, “Intermediate Harvest” (IH), was completed through the FBC. To access the feasibility and potential of this novel process, we first conducted a small-scale screening to identify the preferential time point for the complete media exchange. The influence of the media exchange was investigated during the three main cultivation stages (exponential, stationary, and death phases). The most promising approach was then scaled-up and further optimized to enhance the use of the reactor volume and to study the impact of the FBC separation on the cellular growth kinetics and volumetric productivity of the process.

### 3.1 Small-scale screening

**FIGURE 2:**
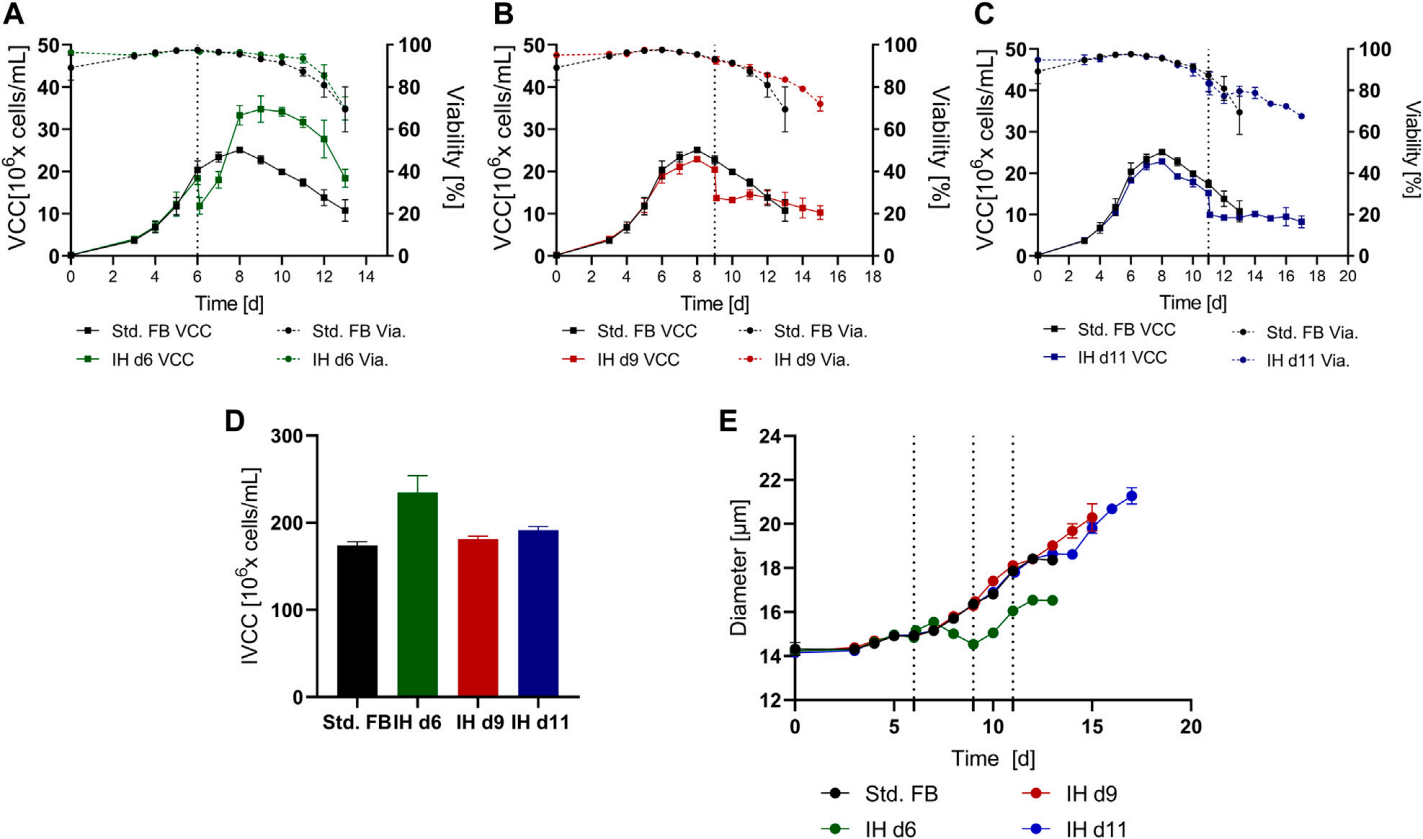
Cultivation results over time for a standard fed-batch (std. FB) cultivation with and without a complete media exchange at different time points. **(A)**, **(B)**, and **(C)** Viable cell count (VCC) and viability (Via.) over time for a media exchange on day 6 (IH d6; *n* = 3), day 9 (IH d9; *n* = 3), and day 11 (IH d11; *n* = 2), respectively, compared to the std. FB (*n* = 3) cultivation; the respective time points of media exchange are indicated by the dotted line. **(D)** IVCC values for all cultures over the total cultivation period. **(E)** Cell diameter over time for each cultivation.

Pilot studies were conducted in the small-scale system to determine the preferable time point for the media exchange over the course of the cultivation. For the IH step, a standard tube centrifuge and manual media exchange were utilized. Three different time points were selected for the IH step that matched, respectively, the exponential phase on day 6 (IH d6), the stationary phase on day 9 (IH d9), and the death phase on day 11 (IH d11) of the culture. The results for the viable cell count (VCC) and cell viability (Via.) for each approach compared to the standard FB (std. FB) without media exchange are shown in Figures 2A–C. Consistent for all cultures subjected to an IH, a clear drop of VCC consecutive to the media exchange is visible. This can be explained by the utilized media exchange method, including manual pipetting and a wash step, which resulted in respective cell recovery rates of 64.2% (IH d6), 66.6% (IH d9), and 65.7% (IH d11). The data show that depending on the cultivation phase, the performed IH has a different impact on cell growth, viability, and process duration. The IH on day 6 resulted in continued growth, an increased peak VCC (+38.7%), and overall higher cell counts in the late phase of the cultivation than the std. FB (Figure 2A) were visible. The cultures with an IH at the respective later time points (Figures 2B, C, respectively), showed no increase in cell density after the IH but an elongated cultivation time of +2 days (IH9) and +3 days (IH11). The selective effect for the early media exchange is further highlighted by the calculated IVCC value (Figure 2D) which is increased for the IH on day 6 (+34.7%) but only slightly impacted for the IH on d9 and d11 (+4.1% and +9.7%, respectively) compared to the std. FB.

Interestingly, the IH on day 6 did not alter the viability profile over time in comparison with the standard approach.

Furthermore, the cell diameter of each approach is shown in Figure 2E. All approaches showed a similar increase in cell diameter over the first six cultivation days, which could be expected due to the same process conditions up to this time point. After the IH on day six, this culture initially showed a decrease in cell diameter. Interestingly, after two days, the diameter started increasing again at about the same rate as the standard culture, resulting in a constant difference of around 2 µm between the IH d6 approach and the std. FB culture. IH d9 and IH d11 cultures showed no decrease in cell size directly after the IH, and the growth of cell size continued at a rate similar to the standard culture over the process time. Therefore, as a consequence of the respective longer process durations of the IH d9 and IH d11, an increased diameter of 2 μm, respectively, 3 μm in comparison to standard culture could be observed for the final day of the cultivation.

**FIGURE 3:**
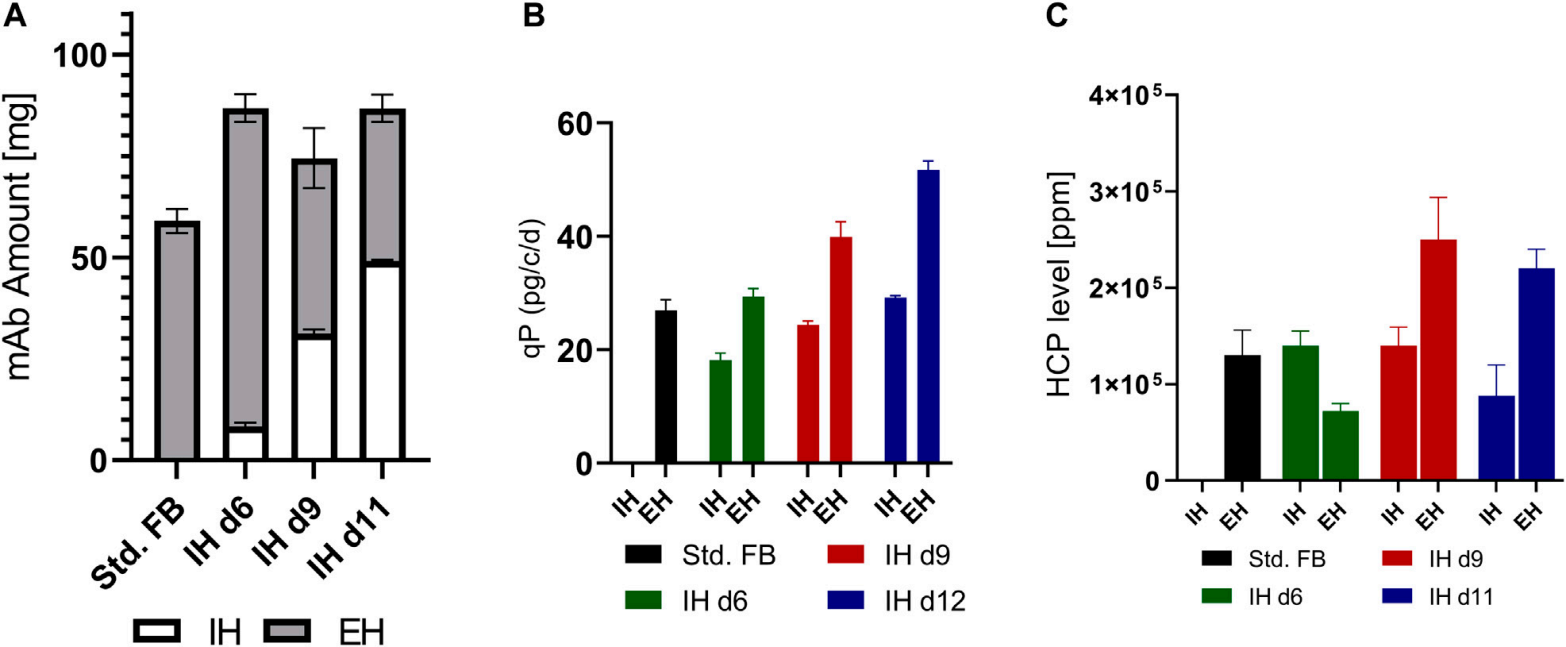
Supernatant analysis for the conducted small-scale screening at the respective time points of the intermediate harvest (IH) and the end harvest (EH). **(A)** Cumulated amount of mAb for each approach overtime in the small-scale screening. **(B)** Mean cell-specific productivity for the first cultivation phase before IH (IH) and for the second phase between IH and EH (EH). Productivities for the control FB were calculated over the whole process duration until the EH. **(C)** Ratio of HCP content to expressed mAb for each approach at the time point of IH and EH.

Subsequently, the produced mAb and impact on process impurities of the different approaches were studied. For that, the impact of media exchange on the total mAb amount, cell-specific productivity (qP), and host cell protein level was investigated. As displayed in Figure 3A, a significant increase in product amount over the whole process duration (cumulative mAb amount for IH + End Harvest (EH)) could be achieved for all IH approaches. To correct for the different vessel volumes caused by the fact that feeding is coupled to the viable cell amount, the total amount of produced mAb was calculated. Compared to the standard FB, an increase in mAb titer of 47.2% for IH d6, 26.9% for IH d9, and 50.0% for IH d11 was observed. As expected, the mAb concentration ratio between IH and EH was strongly in favor of the EH for IH d6 (10:90%; 0.66 g/L: 5.8 g/L), while for the late IH approaches, the ratio shifted to 42:58% (2.4 g/L: 3.2 g/L) for IH d9 and 57: 43% (3.8 g/L: 2.9 g/L) for IH day 11. This observation could be expected as the mAb amount is impacted by the IVCC in the respective cultivation parts. Thereby, the overall increase in mAb amount matches with the enhanced IVCC values for IH d6 cultures. Interestingly, the prediction of matching IVCC values with increase in the mAb amount cannot be applied to IH d9 and d11 cultures, which indicates another underlying factor.

To further visualize this observation, we calculated the qP for each approach up to the IH and afterward until the EH (Figure 3B). The calculations for IH d6 reveal decreased qP for the first 6 days and increased qP after the IH process. The cultures of IH d9 and IH d11 show an even stronger increase in qP in the second cultivation phase after media exchange.

The increase in volumetric productivity either through increase in cellular productivity or cell volume or both is the primary goal for process intensification during the upstream process. Nevertheless, it is crucial for the following downstream operations that process impurities remain within certain boundaries and that the product:impurity ratio is maintained or even increased.

Therefore, to further highlight the potential of the new technology, the impurity content after each harvest cycle was measured to get insight into the supernatant composition and thus in the feed stream subsequently entering the first downstream unit operation. The respective ratio of host cell proteins (HCP) to produced mAb was calculated in ppm (Figure 3C). The HCP level showed an increase of impurities for the complete media exchange in the later stages on d9 and d11, respectively. The lowest HCP impurity level was achieved at the EH with IH d6 cultures, which was 44.6% lower than the standard FB. All further data for the small-scale screening are stated within the Supplementary Figure S1.

Overall, the complete exchange of media showed two distinct impacts on the small-scale cultivation dependent on the IH time point. The supply of fresh media in the exponential phase of the cultivation resulted in increased peak VCC, IVCC, and mAb amounts along with a similar or even reduced impurity level, while the process duration remained similar as that of the standard FB. The exchange of the media in the early or late stationary phase showed no further cell proliferation, a reduced loss of viability over time, and an increase in cell-specific productivity, but also an increase in HCP impurities. For further studies, the IH in the exponential phase was selected as this cultivation approach combined the highest mAb amount (Figure 3A) with a similar or even reduced level of HCP impurities (Figure 3C) and no extension of the overall process duration (Figure 2A). Therefore, the IH at day 6 was considered the most promising strategy for translation into larger scale.

### 3.2 Scale-up

In the next experiment, the IH process was scaled-up to utilize a full-scale FBC and investigate the cellular responses to the operation as well as show the feasibility and optimization of the developed process. The process at 5-L bioreactor scale was conducted for 6 days, then a complete media exchange (IH) was performed using the FBC, and recovered cells were transferred into a suitable 250-mL downscale model. To test the feasibility of the small-scale process, a duplicate reactor set (IH 25) was inoculated from the 5 L reactor comprising similar cell concentrations before and after the IH operation, taking into account the high recovery (>93%) of cells using the FBC system (Saballus et al., 2021). In addition, an optimized approach was conducted in this study with increased cell densities after media exchange (IH opt.). Thereby, the optimized approach comprised the idea to increase the initial filling volume of the reactor from 3 L up to 4 L, leading to an increased fill volume on day 6. In the IH operation, the volume could be decreased by the functional principle of the FBC (Ko and Bhatia, 2012), enabling further feed of the cells and increased cell concentration in the second process. This approach optimized headspace utilization over time in the reactor.

**FIGURE 4:**
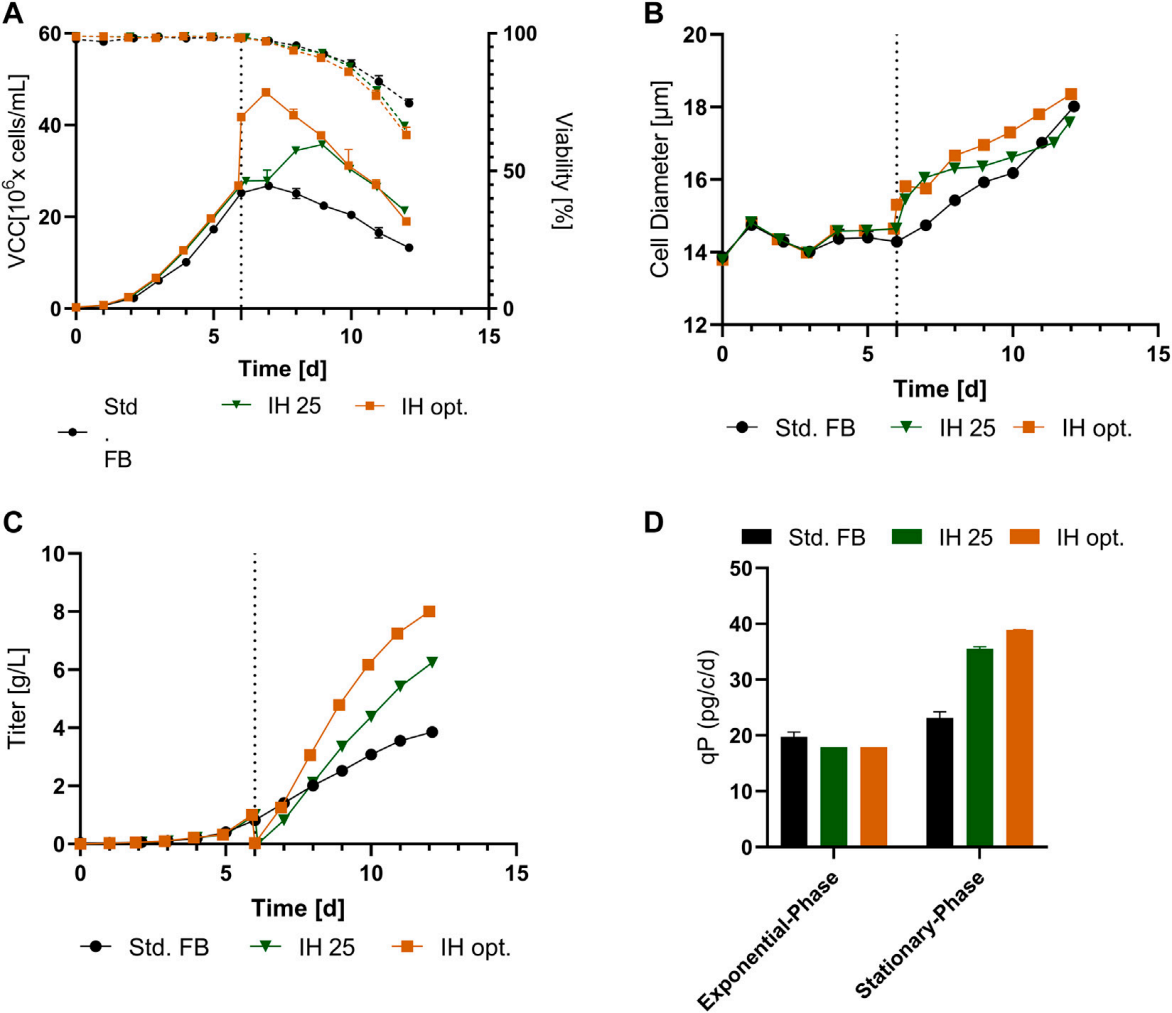
Results for cultivation transferred from a 5-L reactor to a 250-mL downscale model after an IH operation. **(A)** VCC (solid line) and viability (dotted line) profiles over time for standard FB, the IH approach reinoculated at 25 × 10^6^ cells/mL (IH 25), and the optimized IH process (IH opt.) with *n* = 2 for each. **(B)** Cellular diameter for all approaches overtime. **(C)** mAb titer (g/L) over the course of cultivation. **(D)** Cell-specific productivity (qP) in pg/c/d for the exponential and stationary phases for each of the conducted experiments.

The VCC and viability profiles for both processes are visible in Figures 4A. The cultures show a comparable growth pattern up to day 6, comprising the media exchange, as visible for the std. FB. Following the media exchange, IH 25 cultures showed a second proliferation phase visible from days 7 to 9, leading to a peak density of 35.8 × 10^6^ cells/mL, an increase of 34% in comparison to the std. FB. The IH opt. process showed an intended sharp increase of VCC up to 41.7 × 10^6^ cells/mL after the IH process, caused by the concentration of the cells with the FBC operation. Subsequently, a small proliferation phase is visible, leading to a peak cell density of 47.1 × 10^6^ cells/mL on day 8 (+76.4%). Interestingly, for both approaches, no impact on the viability directly after IH was visible; however, a slight decrease in viability for the last day of the process compared to the std. FB was observed (Figure 4A). Furthermore, the cellular diameter, visible in Figures 4B, shows an increase for both cultures processed by the FBC operation consecutive to the media exchange. However, this effect diminishes over the duration of the process, and the values return to the level of the standard cultivation for the IH 25 cultures from day 10 and for the IH opt. on the last day of the cultivation. The titer curves (Figure 4C) show a similar trend for all cultivations in the first 6 days, as expected. The sharp titer drop in the IH cultures on day 6 represents the media exchange and harvest of the produced mAb by the FBC. Afterward, the mAb titer in the IH reactor increased in comparison to the standard process with the sharpest increase for the IH opt. culture, resulting in an increased final mAb concentration of 6.2 g/L for IH 25 and 8.0 g/L for IH opt. compared to 3.9 g/L for the std. FB. In addition to these, cell-specific productivity was calculated for the two separated process phases: the exponential growth phase (up to day 6) and stationary growth phase (from day 6). The respective data are plotted in Figure 4D for all approaches. For the std. FB process, the cell line used in this project reached a qP of around 21 pg/c/d, with a negligible increase in the second phase (stationary phase) of the process. As expected, for the IH cultures, a similar qP compared to the standard culture could be observed (≈18.8 pg/c/d) during the first 6 days, that is, the exponential phase. Interestingly, the cell-specific productivity increases for IH 25 as well as IH opt. strongly after the IH operation. Further process parameter for the cultivation transferred from the 5-L reactors to the 250-mL downscale model are stated within the Supplementary Figure S2.

**FIGURE 5:**
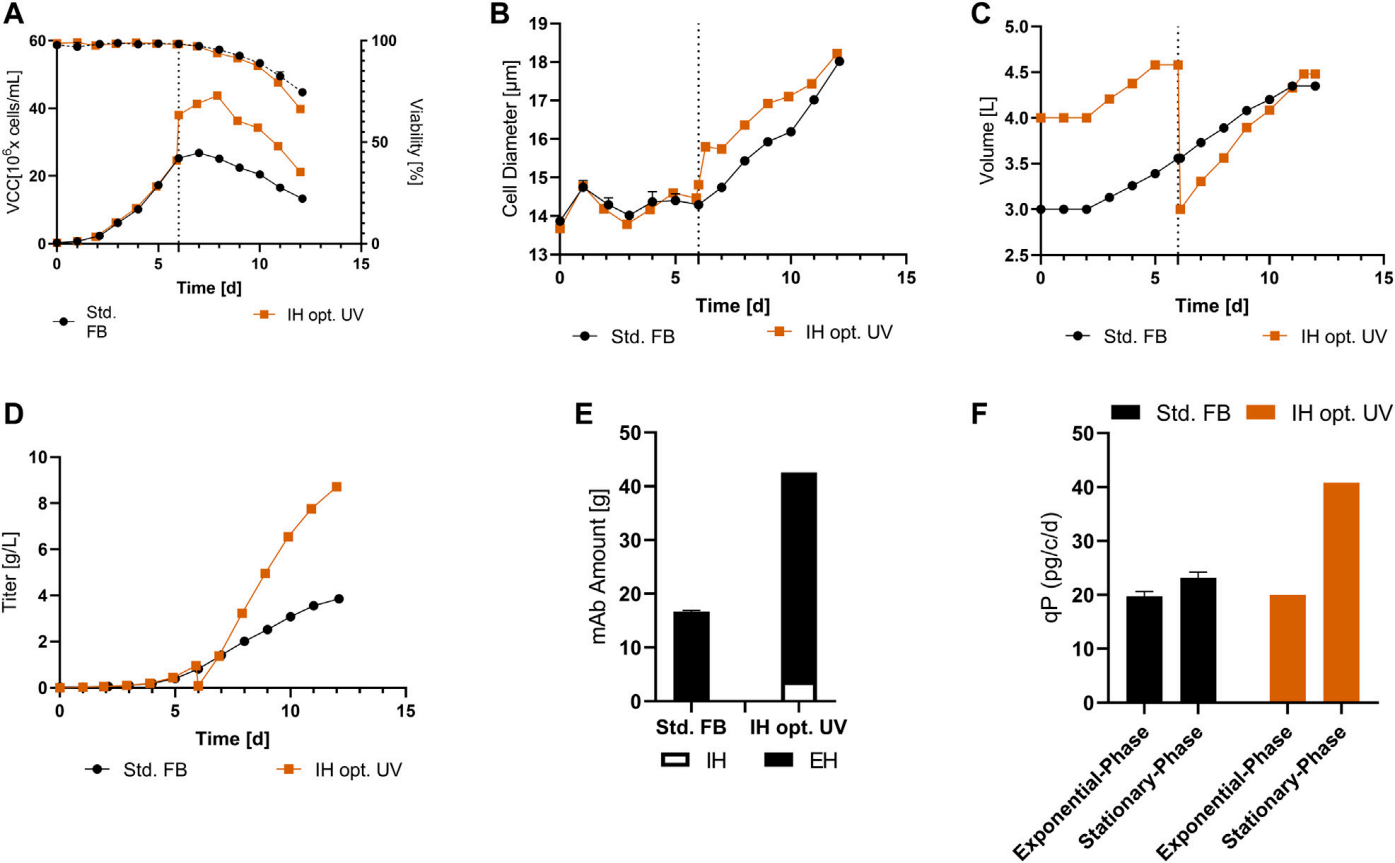
Cellular and expression parameters for the standard FB (*n* = 2) and the optimized IH in a UniVessel cultivation (IH opt.; *n* = 1). **(A)** VCC (solid line) and viability (dotted line) for both approaches. **(B)** Diameter for over the course of cultivation. **(C)** Filling volume overtime for both experiments. **(D)** Titer overtime in (g/L) for std. FB and IH opt. **(E)** Total amount of produced mAb from IH and EH (end harvest) for each approach. **(F)** qP (pg/c/d) separated for the exponential phase (up to day six) and stationary phase (from day six to the end) for both experiments.

To validate the improved process, a full UV cultivation was conducted with the optimized IH process (IH opt.). The data for VCC and viability are displayed in Figure 5A and show similar curve progression as in the IH opt. in the former cultivation. The VCC increased strongly at day 6 due to the concentration obtained with the FBC, followed by a small proliferation phase with a total peak cell density of 43.7 × 10^6^ cells/mL (+63.6%) at day 8. Again, the viability showed no decrease after FBC operation, and analogous to the former cultivation, there was a small differentiation in the last day compared to the std. FB. In Figure 5B, a strong increase in cellular diameter at the IH is visible in comparison to the std. Fb, which is continued to the end of the process. As discussed, before, the strong increase in cell concentration was enabled by the FBC and resulted in a decrease in volume from around 4.5 L to 3 L at day 6 (Figure 5C). This decrease does not only result in a rapid increase of the VCC at the respective day but also results in an increase in the utilization of the reactor volume in the first days and enables the increased feeding after IH operations. Interestingly, comparable filling volumes at the end of the cultivation were reached with the IH opt. and std. FB. To further compare the IH opt. 5 L bioreactor with the std. FB approach, the mAb expression levels were determined. The cultivation fully conducted in the 5-L reactor showed a similar progression to the former cultivation overtime for the titer (Figure 5D) and reached 8.7 g/L at the last day of cultivation. Taking into account the mAb harvest from the first purification during the IH process, the total amount of produced antibody was calculated for the approach. Thereby, both harvests of the IH opt. were added, resulting in a total amount of 42.5 g in comparison to 16.7 g for the standard approach, which represents a total increase of 154% as displayed in Figure 5E. This increase in mAb production is also shown for the space-time-yield (STY) with an increase from 0.27 g/l/d for the std. FB to 0.72 g/l/d for the IH opt. cultivation. Furthermore, the qP values of the two respective processes were calculated (Figure 5F), and they reveal a similar pattern to the first set of experiments with a strong increase in cell-specific productivity in the second part of the cultivation for the IH opt. up to 40.8 pg/c/d. The mass balances of the FBC operation step were determined, which showed high recovery rates for viable cells (99%) and the produced mAb (94.5%). Moreover, the media consumption by the FBC operation could be limited to 1.3 RV for the complete exchange. The overall media consumption could be calculated and increasing from 52 g/L (std. FB), regarding the media powder used per bioreactor volume, to 112 g/L an increase of 115% for the IH opt. approach. An extended set of process parameter for the cultivation solely in the 5-L reactor is presented in the Supplementary Figure S3.

**FIGURE 6:**
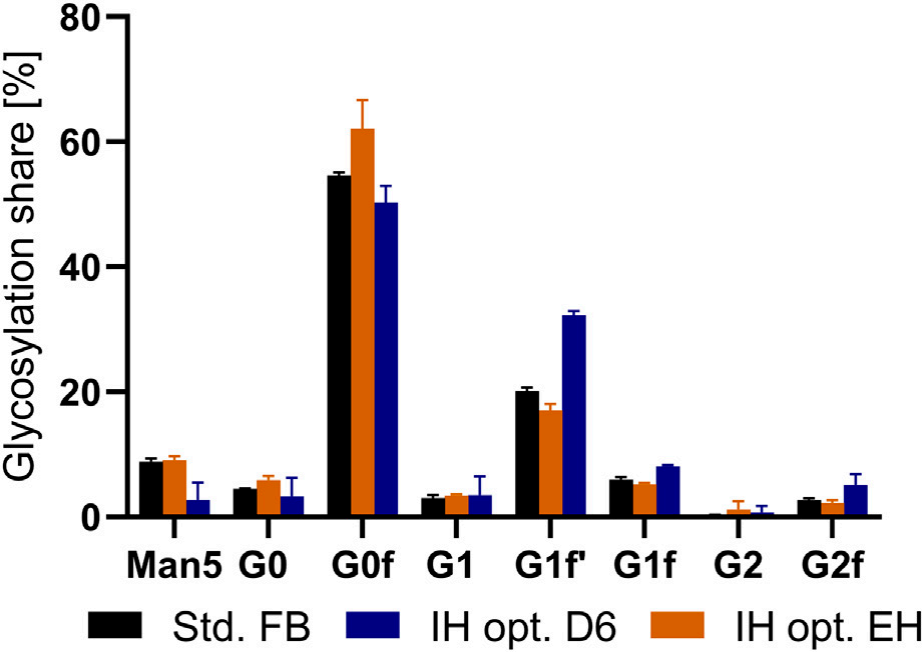
Glycosylation distribution in percentage of expressed mAb from a standard fed-batch (FB) process, and the optimized intermediate harvest (IH opt.) at day six as well as the end harvest (EH).

An increase in volumetric productivity is the key goal of process intensification. However, an important criterion is maintaining the critical quality attributes (CQAs) consistent between standard and novel processes. For mAbs, a typical attribute to assess product quality is the percentual glycosylation share for the produced antibody (Reusch and Tejada, 2015). Therefore, to determine possible impact on product quality, we assessed the glycosylation pattern of the produced antibody from a std. FB as well as all IH opt. processes due to the preferential characteristics. As can be seen from Figure 6, the glycan structures for the standard approach as well as the end harvest of the newly developed IH opt. process remained similar, with only a slight increase in G0F and a decrease in G1f for the IH opt. cultivation. Interestingly, this deviation is considered to be only minor and not visible for the other investigated glycans. However, the purified mAb from the intermediate harvest step shows small alterations compared to the standard process. In detail, a decreased share of mannose 5 and an increase in fractions of galactosylated glycan forms (G1F′ and G2f) could be detected. Nevertheless, the amount of IH antibodies during the IH was only a small fraction (10%) of the total harvest compared to the final harvest.

In summary, the data from the small-scale screening could be re-evaluated in regard to cellular and product-specific parameters in the scaled-up system (IH 25), indicating that the small-scale system is suitable to predict trends in the cultivation. Furthermore, our results suggest that the utilized FBC allows for high mAb as well as cell recovery rates and does not impact cell viability. Finally, we could show that the novel IH process (IH opt.) allowed for an impressive overall increase in productivity of up to 154% with almost similar CQAs and within the same cultivation duration as the standard process.

## 4 Discussion

In this study, a novel strategy for intensification of common FB processes by applying a complete media exchange during the cultivation was developed. To explore the overall potential of such an intensification step and to determine a suitable time regimen, a small-scale screening was conducted using a semi-perfusion method to mimic the IH. Afterward, the approach with the most promising characteristics was scaled-up by implementing the FBC to investigate the influence of this separation method with the single-use FBC and test the feasibility of the new designed process. In a second step, this process was optimized to further enhance productivity and characterize product quality attributes.

### 4.1 Evaluation of cellular responses and critical quality attributes

During the small-scale screening study, we were able to show that depending on the time point of media exchange, different process parameters are influenced. While a media exchange during the exponential growth phase leads to prolonged proliferation of the cells and thereby an increase in peak cell densities, the exchange during the stationary phase does not impact cell concentrations, instead it results in sustained cell viability. This also resulted in an extended process duration for the late media exchange, while the early IH resulted in a process with a similar process time as the std. FB. In general, an impact on cell growth or cell viability can be expected since the media replacement has two distinct consequences: First, new fresh nutrients are supplied to the cells to support proliferation and maintain the cellular state. Second, growth-inhibiting molecules, which accumulate in the supernatant through cellular activity or necrosis, are being removed. Common growth-suppressing molecules are linked with the cellular metabolism, for example, energy metabolism lactate (Lao and Toth, 1997), amino acid metabolism [ammonia (Ozturk et al., 1992), formate (Ihrig et al., 1995)], or the nucleotide metabolism [ADP, AMP (Carvalhal et al., 2003)]. Considering the different cellular states in which the media exchange was conducted, it can be reasonably assumed that the impact on cellular behavior would differ. Accordingly, the media replacement during IH day 6, where the majority of cells are dividing, resulted in further proliferation of cells by removing inhibiting factors and supplying fresh nutrients. A similar effect is utilized in perfusion cultivations, where a continuous media exchange is implemented (Walther et al., 2019). In contrast, the exchange of media in the stationary phase triggers a different cellular behavior with prolonged viability and without proliferation since the majority of cells have already transitioned into the non-dividing state. The transition from the exponential to the stationary phase is not fully analyzed up to this time point but is associated with the accumulation of inhibiting molecules in the supernatant, resulting in several intracellular changes starting at the transcriptional level (Hernández Bort et al., 2012; Sha et al., 2021), thereby likely impacting the metabolic flux in different pathways, for example, pentose phosphate pathway and TCA cycle (Sengupta et al., 2011; Templeton et al., 2013), which is also shown by a change in the holistic cellular behavior like cell size increase (Pan et al., 2017). The overall changes in the cell culture at transition into the stationary phase were permanent for our conducted studies, even with supply of fresh media. Consequently, no further proliferation could be detected, but instead, an elongated viability profile was observed in the respective cultures, which could be caused by the removal of inhibiting molecules in the supernatant released by cellular activity or disrupted cells. Example for these apoptotic stimulating molecules in mammalian cells are extrinsic signaling stimuli like FasL or TNFα with respective membrane receptor proteins (TNF-R1 and Fas) (Curtin and Cotter, 2003; Meshram et al., 2010), or metabolic derived products, for example, dimethylarginine and methylglyoxal (Chaplen, 1998; Jiang et al., 2006). The media matrix replacement could therefore be responsible for the elongated process durations. Nevertheless, to prove this theory, more experiments need to be conducted with different cell lines, which was not the main focus of this work.

In addition to the impact on cellular growth and viability, the exchange of media also resulted in an increased productivity of the used CHO cell line. This behavior is visible for the cell-specific productivity which could be increased up to 76.4%, representing the IH d11 approach in the small-scale trials. Furthermore, the increased HCP values observed during the small-scale screening (IH d9 and IH d11) support the assumption of a general increase in protein expression after media exchange. These increases in expression are accompanied by an increase in diameter (up to + 3 µm) in the respective cultures. Thereby, increased diameter could be linked to enhanced cell-specific productivity by previous investigations (Lloyd et al., 2000; Pan et al., 2017) which could partly explain the measured data. Interestingly, small-scale culture IH d6 showed no alternating cell-specific productivity and a decrease of HCP values with a simultaneous decrease in diameter (−2 µm) in contrast to the std. FB (Figures 2D, 3B). Especially, the constant mAb expression level for these cultures was surprisingly due to previous investigated relation between cell sizes and qP which would entail a decrease in qP. Therefore, a decrease of qP in this culture would be suggested as triggered by the decrease of diameter. The constant expression levels thereby indicate a further underlying mechanism boosting the cell-specific productivity of the cells after the media exchange and should be the subject of further investigation. The subsequent scale-up of the process with the IH25 culture confirmed the trend of the small-scale screening, with small alterations to specific parameters. This scale-up will be further discussed in Section 4.2.

In addition to these, the IH opt. cultivation showed a different cellular characteristic from the previous IH operation (IH 25). Thereby, the IH opt. process caused a sharp increase in VCC after the IH operation, which was caused by a strong volume reduction of around 1/3 in the reactor. This volume reduction served two goals: first, it increased the headspace utilization of the reactor overall in the first six days, and second, essential headspace was cleared to feed the cells in the second part of the process. This sharp increase of VCC thereby alters the trend of the cultivation visible in the comparison between IH opt. and IH 25 (Figure 4). First, the proliferation phase of these cultures was diminished in comparison to IH 25 cultures. However, enhanced peak cell densities could be reached with this approach mainly due to the strong concentration of the cells in the FBC operation. An explanation for this diminished proliferation phase could be the strong increase of inhibiting molecules resulting from the high cell count in the first days after IH operation, or a sharp decrease of a nutrient component resulting in a fast transition into the stationary phase of the culture. Furthermore, the cellular diameter increased overtime in comparison to IH 25 cultivation mainly in the latest days (Figure 4B; day 9–12). Overall, both processes show a sharp increase in diameter after IH operations, which will be further discussed in Section 4.2. Nevertheless, the increased diameter overtime can be caused by the diminished proliferation phase of the IH opt. cultures, mainly due to the cell size increase in preparation for the cell division, which seems to be interrupted for this culture. This explanation is supported by the cell size decrease of culture IH d25 and IH d6 which shows a reduction of cell size in the proliferation phase (Figures 2E, 4B). Further studies need to be conducted to analyze the reasons for this behavior, which was not the focus of this work. Nevertheless, similar cultivation times and increased mAb titers were achieved in comparison to the IH 25 process. This leads to the conclusion that the IH opt. process is the more favorable application of these two processes.

In summary, our data show that the novel IH strategy represents a very efficient and robust method for process intensification because volumetric productivities were increased significantly. However, in addition to product yields, product quality is highly relevant for biopharmaceuticals. Thereby, the product quality can impact the therapeutic efficacy, toxicological profile, and *in vivo* lifetime. To demonstrate that the process is applicable for the production of therapeutic proteins and can be developed into a commercial process, the critical quality attributes were investigated to verify consistent posttranslational modification of the product. The glycosylation pattern of the produced mAb was analyzed for the IH opt. as well as for the std. FB. The results show a similar glycan structure between both terminal harvests in addition to the G0F values, with a minor increase in the IH operation. Due to the minor increase as well as the similar pattern for the rest of the glycans, possibly no differences in product quality can be expected. However, in contrast to the final harvest (EH), the glycan analysis for the IH showed a slight shift toward higher galactosylated forms. Glycosylation is influenced by a variety of different variables, for example, concentration of certain molecules, pH, and DO (Hossler et al., 2009). One such molecule is ammonia which has shown to negatively influence the galactosylation rate of antibodies with increasing concentration (Gawlitzek et al., 2000). Thereby, levels for ammonia increased from the IH to the EH in the intensified process from around 2.22 mM–12.3 mM (data not shown), which could explain the differences in the glycosylation profile between both harvests. These alterations of the galactosylation can influence the potency of the produced antibody. Depending on the antibody mechanism of action, complement-dependent cytotoxicity (CDC) or antibody-dependent cell-mediated cytotoxicity (ADCC), a higher share in galactosylation can increase the potency of the product (Boyd et al., 1995; Kanda et al., 2007). Thereby, the observed alteration of the glycosylation could potentially benefit the overall product efficacy, but this needs to be further investigated and will most likely depend on the individual mAb compound. One possibility would be a combination of both harvests, but further tests are necessary to confirm the safety of this approach. Nevertheless, the amount of produced antibody in the IH opt. UV only takes a small share to the total amount of produced protein, so an omission of these would reduce the increase of productivity neglectable to +132%.

### 4.2 Evaluation of scale-up and separation operation

Key elements for the fast development and implementation of novel process intensification strategies are adequate scale-down models. In this work, a small-scale mimic process was conducted to evaluate the behavior of the cells during the complete media exchange. To evaluate the transferability between the two scales, culture IH d6 (small-scale) was compared to the IH 25 (scale-up). Thereby, the scaled-up cultivation showed similar process characteristics as observed in the small-scale study, including comparable peak cell density (≈3% difference), a second proliferation phase after IH, and comparable titers at the end of the process (IH d6 = 5.7 g/L; IH 25 = 6.2 g/L). However, in contrast to the small-scale cultivation, a short lag-phase between days 6 and 7 with a simultaneous increase in diameter could be observed during upscaling. As discussed previously, an increased diameter can positively affect the cell-specific productivity, which furthermore could explain the qP differences from the small-scale IH d6 culture [29.3 pg/c/d (Figure 3B)] to the IH25 culture [35.5 pg/c/d (Figure 4D)]. A possible reason for the different cellular behaviors that were mainly observed after media exchange could be caused by the different applied separation methods. It can be assumed that cellular behavior in a standard centrifugation process differs from that in a FBC operation with a counteracting centrifugal and flow force. However, the actual underlying mechanisms resulting in the observed effect, especially the notable increase in cellular productivity, need to be analyzed in more detail and should be the subject of future investigations. Another visible difference can be noticed in the reinoculation VCCs for both cultures at day 6. The differences were caused by the small-scale separation method with limited cell recovery rates; this could be minimized in the 5 L scale operation due to efficient separation by the FBC. In small-scale experiments, falcon tubes and a centrifuge were used for separation, and the cells were washed twice, which decreased the recovery rate to around 64%, mostly due to unavoidable dead volume. Meanwhile, the clarification by the single-use FBC showed a high recovery rate of 99% of all cells. This led to the discrepancy in reinoculation VCC after the IH operation between small-scale and scale-up cultures. Overall, the small-scale screening system seems to be capable of predicting advantageous process parameters, even with the differences in separation method, and showed sufficient predictability for larger-scale cultivations.

Another essential element to the established new process is the gentle, sterile, fast, and scalable separation of the cells from the supernatant. In this work, a single-use FBC was utilized as a separation tool. Interestingly, the viability profiles for the IH and standard cultures showed a similar trend overtime visible in Figures 4A, 5A despite the small deviation at the end of the process. This indicates a non-disruptive separation of the cells without any negative impact on cell viability or performance throughout the rest of the process. Interestingly, the cell diameter for all processed cultures showed a diameter increase consecutive to the IH operation. This increased diameter showed no impact on the viability of the cells and possibly even increased the cell-specific productivity for the respective cultures, as discussed beforehand. Therefore, this unintended impact on cellular behavior can be assumed to be a beneficial feature for the overall process. An explanation for the increased diameter of the cells after IH operation could be the impact of the FBC separation methods with different opposing forces compared to common centrifugation. In addition to these, the FBC operation resulted in high cell (99%) and mAb (94,9%) recovery rates, as already reported by others (Ko and Bhatia, 2012; Kelly et al., 2016; Saballus et al., 2021). Overall, the established cell and product separation *via* the FBC showed no increased risk for contamination, low restrictions for scale-up, as well as decreased process monitoring in comparison to the continuous filter-based media exchange. These characteristics make the FBC a valuable tool for novel FB process intensifications. Furthermore, our results showed an overall media consumption increase of +115% that was accompanied by an impressive 154% increase in product titer. Taking into account that media is one of the main cost drivers during the upstream process, it can be assumed that these novel processes represent a highly innovative approach for cost-efficient process intensification. However, to fully excess the process potential from an economic perspective, a holistic cost modelling needs to be performed in the future, comprising further cost-influencing factors such as increased needs for consumables and manpower that was not the subject of this study.

## 5 Conclusion

In this study, a novel hybrid process, positioned between perfusion cell culture and classical FB cultivation, was developed. Hence, the impact of a complete and rapid media exchange with a FBC system (IH) during a common FB cultivation was studied. Our results show two distinct effects on the cultivation depending on the time point of the IH. During the exponential growth phase, a significant prolonged proliferation of the cells can be achieved, resulting in higher peak cell densities. In contrast, an IH during the stationary phase results in a sustained arrest of cell proliferation, prolonged high cell viabilities, and a subsequent elongation of the process duration. Considering no significant differences in final product titer and an increased impurity to product ratio for the IH at later time points, the IH during the exponential growth phase was chosen for upscaling. The feasibility as well as small-scale comparability could be demonstrated in a scaled-up system. Moreover, the process could be further optimized in a subsequent step, by increasing the utilization of the vessels and subsequently concentrating the cells after the IH operation. The results revealed that the novel process, enabled by the FBC, allows for an increase in overall product yield per run of +154% mAb in comparison to a std. FB. Overall, this work demonstrates an innovative new method for intensifying commonly used FB operations without the complex integration of a perfusion cell culture system. Moreover, combination of this application with various other intensification methods, such as high inoculation FB, is conceivable due to the simplicity of the IH process. Furthermore, the established process should be fully scalable up to 2000 L scale due to an already existing scale-up version of the FBC system. However, a proof of concept at production scale (e.g., 2000 L) still needs to be conducted, which will be the focus of future studies. Overall, the proposed work opens up several new possibilities to modify the current FB process in order to significantly increase product yields and production rates.

## Data Availability

The raw data supporting the conclusions of this article will be made available by the authors, without undue reservation.
