# Supplementary figures and images for: Boosting Productivity for Advanced Biomanufacturing by Re-Using Viable Cells
Source: Front Bioeng Biotechnol. 2023 Feb 16;11:1106292. doi: 10.3389/fbioe.2023.1106292 (PMC9978186; doi:10.3389/fbioe.2023.1106292)

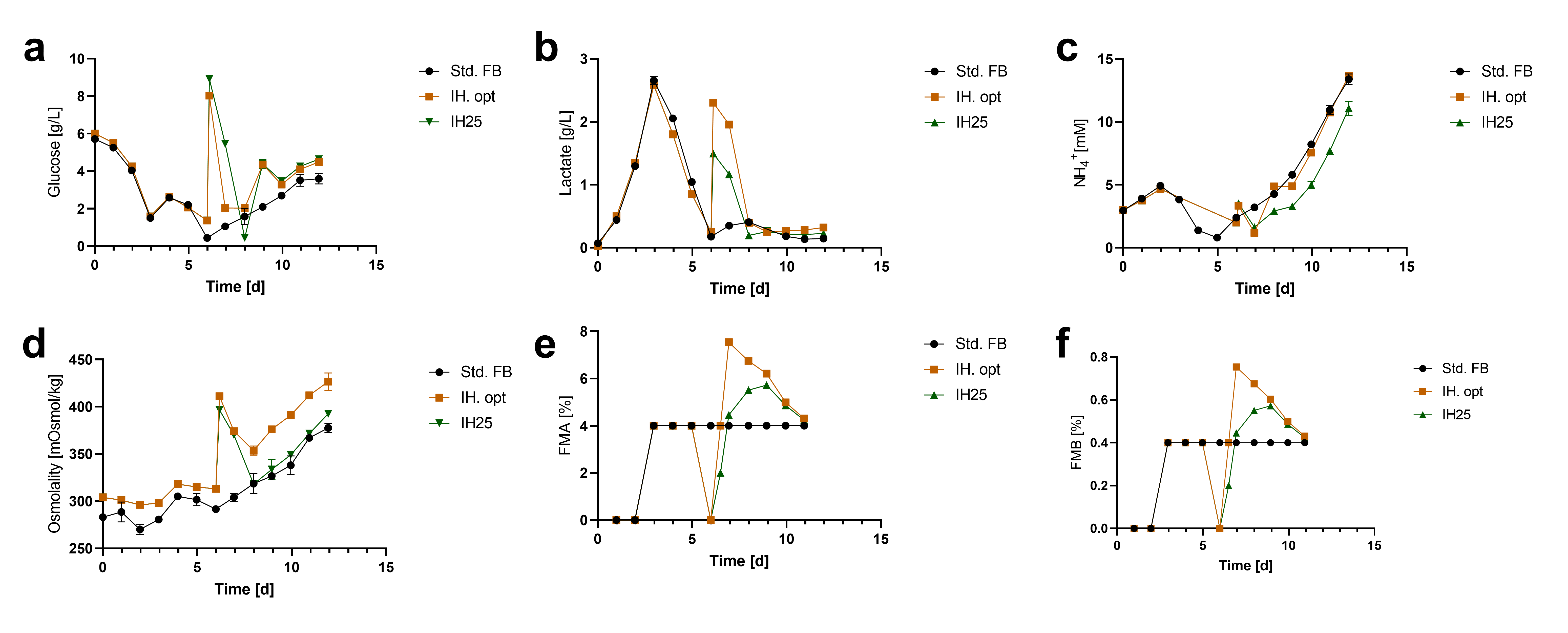

Supplement: Supplementary file 1 [file Image3.TIF]

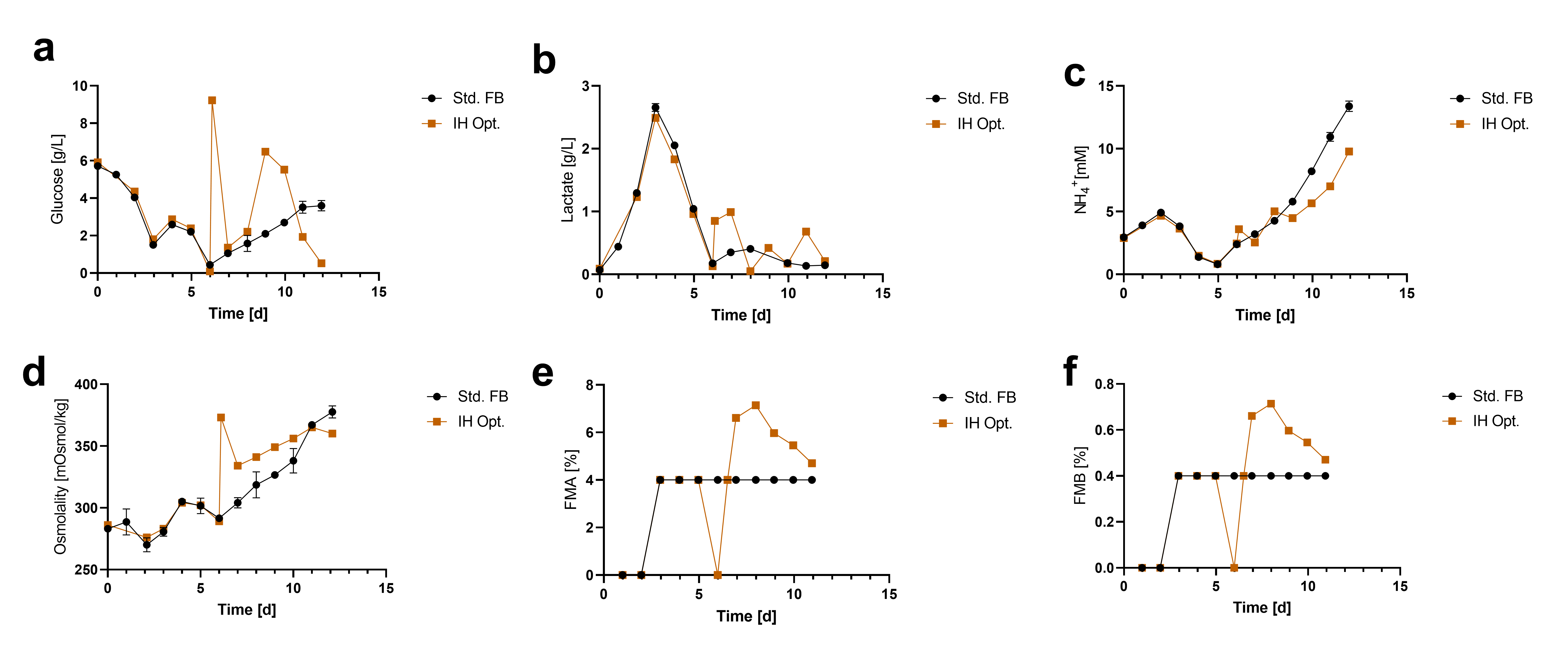

Supplement: Supplementary file 2 [file Image2.TIF]

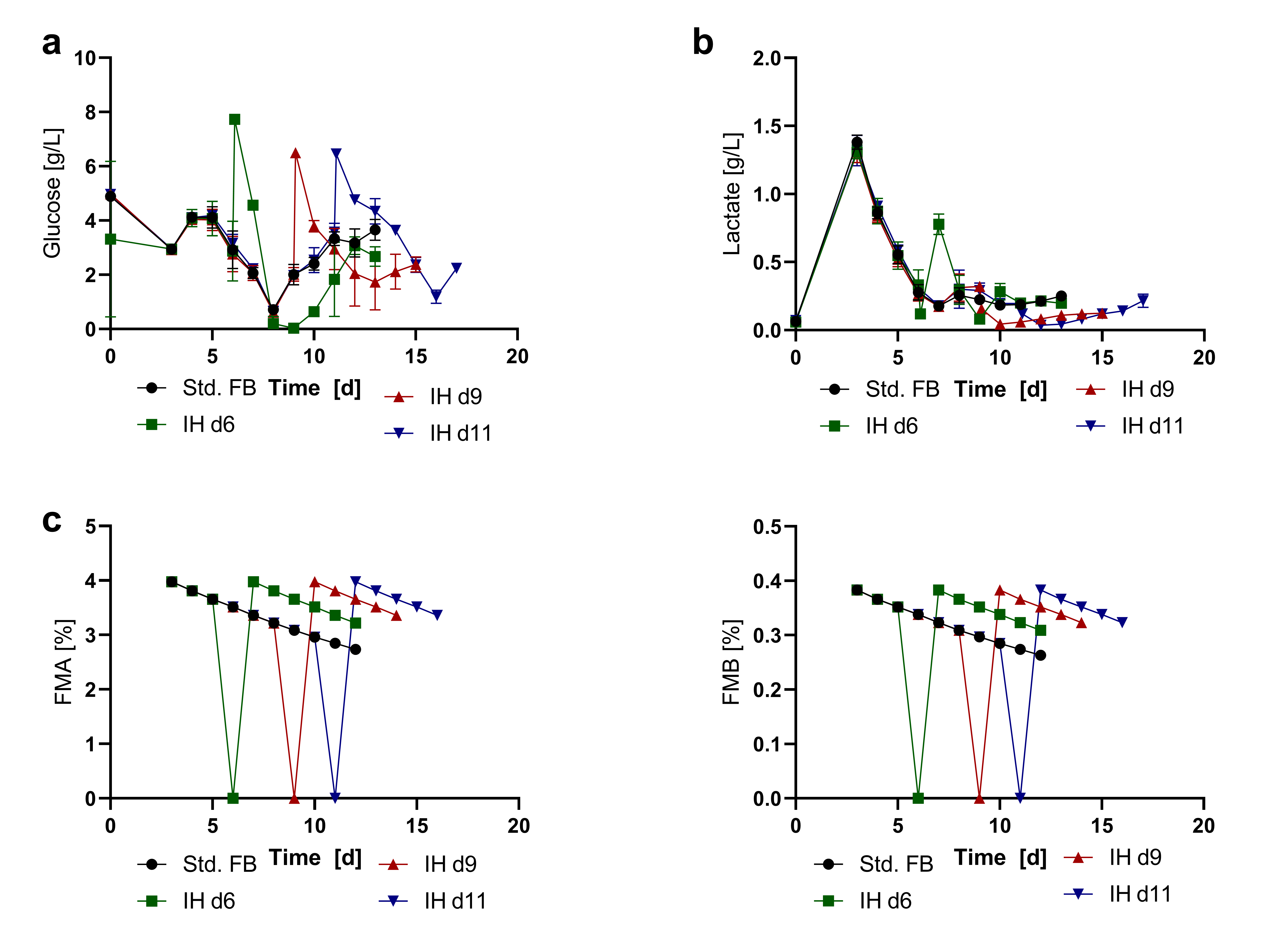

Supplement: Supplementary file 3 [file Image1.TIF]
