# Supplementary material for: Boosting Productivity for Advanced Biomanufacturing by Re-Using Viable Cells
Source: Front Bioeng Biotechnol. 2023 Feb 16;11:1106292. doi: 10.3389/fbioe.2023.1106292 (PMC9978186; doi:10.3389/fbioe.2023.1106292)
Supplement: Supplementary file 4 [file DataSheet1.PDF]

Supplementary:

Additional metabolite data, osmolality profiles and feed percentages, when available, are provided for the cultivations conducted in the previous work.

The Figure 1 shows the mentioned parameter for the small-scale screening.

Figure 1: Metabolite and Feed characteristics over time for a standard fed-batch (std. FB) cultivation with and without a complete media exchange at different time points. Media exchange was conducted on day 6 (IH d6), day 9 (IH d9), and day 11 (IH d11) respectively. (a) Glucose values for the conducted cultivation overtime. (b) Lactate values for the conducted cultivation overtime. (c) Percentage of FMA feed for each day. (d) Percentage of FMB feed for each day.

The Figure 2 shows the mentioned parameter for the scale up comprising a 250 mL downscale model after IH operation.

Figure 2: Results for cultivation transferred from a 5 L reactor to a 250 mL downscale model after IH operation. (a-c) Glucose, lactate and NH_4^+ values for the conducted studies overtime. (d) Osmolality profile for each approach overtime. (e, f) Percentage of feeding for FMA and FMB overtime for each approach.

The Figure 3 shows the mentioned parameter for the UV comparison of the new designed IH process to a standard cultivation.

Figure 3: Results for the cultivation of the standard FB and IH process fully conducted in the UV. (a-c) Metabolite values (glucose, lactete and NH_4^+) overtime for the conducted experiments. (d) Osmolality values overtime for the two different approaches. (e, f) Feeding percentage of FMA and FMB overtime.
